# Dr. Mortimer S. Coon

**Published:** 1905-04

**Authors:** 


Dr. Mortimer S. Coon, of Medina a graduate of the University of Buffalo, 1892, died at his home March 5, 1905, aged 37 years. His death followed an operation for appendicitis. He was a prominent citizen of Orleans county and took an active part in public affairs. He is survived by a wife and one daughter.

